# Preeclampsia complicated by advanced maternal age: a registry-based study on primiparous women in Finland 1997–2008

**DOI:** 10.1186/1471-2393-12-47

**Published:** 2012-06-11

**Authors:** Reeta Lamminpää, Katri Vehviläinen-Julkunen, Mika Gissler, Seppo Heinonen

**Affiliations:** 1 Department of Nursing Science, University of Eastern Finland, PO. Box. 1627, Kuopio, 70211, Finland; 2 Department of Nursing Science, University of Eastern Finland and Kuopio University Hospital, Kuopio, Finland; 3 National Institute for Health and Welfare (THL), Gothenburg, Sweden; 4 Nordic School for Public Health, Helsinki, Finland; 5 Department of Obstetrics and Gynaecology, Kuopio University Hospital and University of Eastern Finland, Kuopio, Finland

## Abstract

**Background:**

Preeclampsia is a frequent syndrome and its cause has been linked to multiple factors, making prevention of the syndrome a continuous challenge. One of the suggested risk factors for preeclampsia is advanced maternal age. In the Western countries, maternal age at first delivery has been steadily increasing, yet few studies have examined women of advanced maternal age with preeclampsia. The purpose of this registry-based study was to compare the obstetric outcomes in primiparous and preeclamptic women younger and older than 35 years.

**Methods:**

The registry-based study used data from three Finnish health registries: Finnish Medical Birth Register, Finnish Hospital Discharge Register and Register of Congenital Malformations. The sample contained women under 35 years of age (N = 15,437) compared with those 35 and over (N = 2,387) who were diagnosed with preeclampsia and had their first singleton birth in Finland between 1997 and 2008. In multivariate modeling, the main outcome measures were Preterm delivery (before 34 and 37 weeks), low Apgar score (5 min.), small-for-gestational-age, fetal death, asphyxia, Cesarean delivery, induction, blood transfusion and admission to a Neonatal Intensive Care Unit.

**Results:**

Women of advanced maternal age (AMA) exhibited more preeclampsia (9.4%) than younger women (6.4%). They had more prior terminations (<0.001), were more likely to have a body mass index (BMI) >25 (<0.001), had more in vitro fertilization (IVF) (<0.001) and other fertility treatments (<0.001) and a higher incidence of maternal diabetes (<0.001) and chronic hypertension (<0.001). Multivariate logistic regression indicated that women of AMA had higher rates of: preterm delivery before 37 weeks 19.2% (OR 1.39 CI 1.24 to 1.56) and before 34 weeks 8.7% (OR 1.68 CI 1.43 to 2.00) low Apgar scores at 5 min. 7.1% (OR 1.37 CI 1.00 to 1.88), Small-for-Gestational Age (SGA) 26.5% (OR 1.42 CI 1.28 to 1.57), Asphyxia 12.1% (OR 1.54 CI 1.34 to 1.77), Caesarean delivery 50% (OR 2.02 CI 1.84 to 2.20) and admission to a Neonatal Intensive Care Unit (NICU) 31.6% (OR 1.45 CI 1.32 to 1.60).

**Conclusions:**

Preeclampsia is more common in women with advanced maternal age. Advanced maternal age is an independent risk factor for adverse outcomes in first-time mothers with preeclampsia.

## Background

Maternal age at the time of first delivery has increased in many Western countries [1]. A woman who is 35 years or older at the time of delivery has been defined as being of “advanced maternal age (AMA)” [2]. In Finland, the mean age of women giving birth has, for a long time, been around 30 years, and the average age in 2009 was 28 years among primigravidas. However, the proportion of parturients aged over 35 in 2009 was 18.7%, and this percentage has been consistently increasing [3].

Several studies have shown an association between AMA and adverse perinatal outcomes as well as an increased risk of certain pregnancy complications. Specifically, women of AMA have an increased risk of gestational diabetes, placenta previa [4-6], preeclampsia [5,7], miscarriage [4], pregnancy-induced hypertension [5] and the need for Caesarean deliveries [8]. Induction of labor [5,9,10], augmentation with oxytocin and assisted deliveries are also known to be associated with women of AMA [10]. Furthermore, increasing maternal age contributes to fetal, perinatal and neonatal death [5]. Women of AMA are also more likely to have been diagnosed with chronic diseases, such as hypertensive disorders and diabetes mellitus, thus further complicating their pregnancies [4,5,7,9,10].

Preeclampsia is a frequent and potentially severe disease that affects about 3-8% of all pregnancies and increases the mothers risk for morbidity [11-13]. Risks associated with preeclampsia include nulliparity, pre-existing medical conditions (e.g. hypertension, diabetes mellitus and anti-phospholipid syndrome), plurality, older maternal age and obesity [11,12]. The aim of this study was to compare pregnancy outcomes in women with preeclampsia aged 35 and over to those with preeclampsia under 35 years of age to examine differences in obstetric outcomes.

## Methods

### Data and variables

The original dataset contained information on 690,555 women and their newborns, for births between the years 1997 and 2008. Eligibility criteria for this analysis included primiparous women with singleton pregnancies diagnosed with preeclampsia without malformations (N = 17,824).

Approval for the study was obtained from the National Institute for Health and Welfare (THL). The data used in this study consisted of information from the Finnish Medical Birth Register (MBR), Hospital Discharge Register (HDR) and The Register of Congenital Malformations. The THL stores information from each of these registers and was responsible for providing all of the data.

The Finnish Medical Birth Register is a population-based registry established in 1987 and is currently compiled by the THL. The MBR includes information on maternal and neonatal birth characteristics and perinatal outcomes for all women giving birth in Finland and all newborns up to seven days of age. The form is filled in by the hospital and sent, typically in electronic format, to the THL [14].

The Hospital Discharge Register was established in 1967 and contains information on all aspects of inpatient care in public and private hospitals and outpatient visits to public hospitals (since 1996) in Finland. The data are sent electronically to the THL by each setting [15].

The Register of Congenital Malformations is controlled by THL and contains data on congenital chromosomal and structural anomalies detected in stillborn and live born infants and terminated fetuses from all healthcare settings in Finland. The Register was established in 1962 and registration of anomaly data began in 1963 [16].

**Figure 1:**
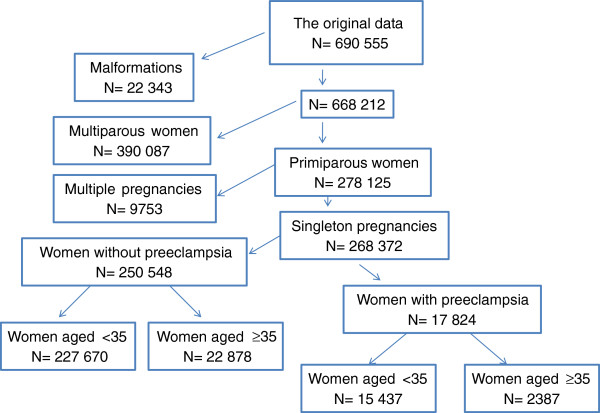
The data flow in the current study222223232323.

We compared women with preeclampsia who were of AMA to women under the age of 35. The study group contained 2,387 women aged 35 and over and the control group included 15,437 women under the age of 35 (Figure 1).

Preeclampsia was defined as repeated periods of blood pressure > 140/90 mmHg accompanied by proteinuria (>0.3 g/day). Participants were identified in two ways. First, we included eligible women based on diagnoses recorded using ICD-10 codes (preeclampsia: ICD-10 codes O11, O14 and with seizures O15). All identified cases of preeclamptic and eclamptic pregnancies were used in the analysis. Second, we identified participants who were hospitalized during pregnancy because of hypertension. This was necessary because, in the MBR, ICD-10 coding of diagnoses during pregnancy did not start until 2004. Some of the women with preeclampsia were hospitalized during pregnancy, but they were not allocated ICD-codes and were recorded as being hospitalized because of hypertension; however, this diagnosis generally identifies those with preeclampsia.

The following definitions were used to record pregnancy outcomes: preterm delivery, before 34 and 37 weeks of gestation; Apgar score <7 at 5 minutes; and small for gestational age (SGA), infants whose sex- and age-adjusted birth weight was below the tenth percentile according to the normal tables for our population [17].

### Statistical analysis

Statistical analysis was conducted using SPSS for Windows, version 17. Statistical differences between the study and control groups were evaluated using chi square-tests for dichotomous variables; continuous variables were analyzed using independent sample t-tests.

All variables used in the binary logistic regression analysis were dichotomous and missing data for any variable were categorized as “no” (=0). Binary logistic regression adjusted for potential confounding factors included prior termination, placenta previa, IVF, fertility treatment other than IVF, smoking, maternal age (in the categories under 35 and 35 and over), maternal diabetes (including both pre-pregnancy and gestational diabetes) and BMI >25 before pregnancy. Because maternal height and weight have been recorded in the data sources only since 2004, logistic regression is presented primarily without BMI (Table 1). To compare the pregnancy outcomes of women with preeclampsia under 35 years of age to those aged 35 and over, odds ratios with 95% confidence intervals were estimated.

**Table 1:** Results of binary logistic regression analysis of women aged under 35 and 35 and over with preeclampsia (* BMI included)

**Outcome**	** <35**	** ≥35**	** Adjusted P**	**Adjusted OR**	**95% CI**
Preterm delivery (before <34 wk)	830 (5.5)	203 (8.7)	<0.000 (* < 0.000)	1.68 (*1.81)	1.43-2.00 (*1.36-2.42)
Preterm delivery (before 37 wk)	2231 (14.5)	457 (19.2)	<0.001 (*0,002)	1.39 (*1.36)	1.24-1.56 (*1.12-1.65)
Low Apgar score (<7) at 5 min	276 (5.5)	52 (7.1)	0.048 (*0.122)	1.37 (*1.30)	1.00-1.89 (*0.93-1.83)
SGA (<90^th^ percentile)	3233 (21.0)	632 (26.5)	<0.001 (* < 0.001)	1.42 (*1.53)	1.28-1.57 (*1.29-1.82)
Fetal death	28 (0.2)	6 (0.3)	0.781 (*0.964)	1.14 (*0.96)	0.45-2.86 (*0.19-4.98)
Asphyxia	1229 (8.0)	289 (12.1)	<0.001 (*0.006)	1.54 (*1.38)	1.34-1.77 (*1.10-1.73)
Cesarean	4928 (31.9)	1192 (50.0)	<0.001 (* < 0.001)	2.02 (*1.86)	1.84-2.20 (*1.60-2.17)
Induction	12115 (78.5)	1697 (71.1)	<0.001 (*0.001)	0.67 (*0.73)	0.61-0.74 (*0.61-0.87)
Eclampsia	157 (1.0)	20 (0.8)	0.279 (*0.752)	0.77 (*1.13)	0.48-1.23 (*0.54-2.34)
Blood transfusion	244 (1.6)	38 (1.6)	0.311 (*0.437)	0.83 (*0.85)	0.58-1.19 (*0.57-1.27)
Admission to a neonatal unit	3650 (23.6)	755 (31.6)	<0.001 (* < 0.001)	1.45 (*1.42)	1.32-1.60 (*1.20-1.65)

## Results

Preeclampsia was identified in 9.4% of women of AMA and 6.4% of women under 35 years of age. The mean (± standard deviation) ages of the control (<35) and study groups (>35) were 26.6 ± 4.2 years and 37.5 ± 2.3 years, respectively. The groups were different in terms of prior terminations, pre-pregnancy obesity (BMI >25), IVF and other fertility treatments, smoking, chronic hypertension and maternal diabetes. Table 2 summarizes the background information for the two groups.

**Table 2:** Background information about women aged under 35 and 35 and over who had preeclampsia

	**Women <35**	**Women ≥35**	
	**(n = 15 437)**	**(n = 2387)**	**P**
Prior termination	3237 (21.0)	812 (34.0)	<0.001
Pregravid BMI >25 kg/m^2^	2201 (41.6)	470 (56.2)	<0.001
Late pregnancy bleeding	215 (1.4)	45 (1.9)	0.062
Placenta previa	29 (0.2)	11 (0.5)	0.009
Fertility treatment other than IVF	274 (1.8)	154 (6.5)	<0.001
IVF	291 (1.9)	184 (7.7)	<0.001
Anemia	91 (0.6)	14 (0.6)	0.986
Smoking	2196 (14.2)	226 (9.5)	<0.001
Not married	7346 (47.6)	1128 (47.3)	0.058
Chronic hypertension	177 (1.1)	49 (2.1)	<0.001
Maternal diabetes	753 (4.9)	221 (9.3)	<0.001

The mean weight before pregnancy in the control group was 69.6 ± 16.3 kg versus 73.9 ± 16.3 kg (<0.001) in the study group. In the control group the mean birth weight was 3179 ± 730.4 g versus 3037 ± 788.3 g (<0.001) in the study group. Pregnancy outcomes for the two groups are compared in Table 1.

Cesarean delivery was found to be twice as likely in women of AMA. The rate of preterm delivery before 34 and 37 weeks’ gestation, low Apgar score at 5 min., SGA, asphyxia and admission to a NICU were also significantly higher in women of AMA. No significant differences between groups were found in rates of fetal death, eclampsia and blood transfusion to mothers.

## Discussion

Women of AMA were 1.5 times more likely to have preeclampsia compared to women under 35 years of age. The main finding of the present study, however, was that among preeclamptic women, adverse pregnancy outcomes were more common in women of AMA.

First, women of AMA were significantly more likely to have preterm deliveries before 34 and 37 weeks and to have SGA- infants the risk increase being 70% in preterm delivery before 34 weeks and 40% in both preterm delivery before 37 weeks and SGA. Second, women of AMA had a twofold increased risk of requiring Cesarean deliveries. Finally, these obstetric risks led to approximately 50% higher frequencies of neonatal asphyxia and 40% of admission of the infant in the neonatal intensive care unit.

Similar to other studies, the women of AMA in this sample experienced more prior terminations and IVF and other fertility treatments [18-20]. On the other hand, they smoked less often than younger women, but they were more often obese and had higher rates of gestational diabetes. Obesity is known to be a risk factor for preeclampsia [12], whereas smoking is a protective factor [21]. The net effect of maternal obstetric history and health behavior is likely to play a minor or moderate role in the entire picture, since some of the effects are beneficial while others are harmful depending on whether the outcome under consideration is the frequency of preeclampsia, premature delivery, fetal growth or neonatal outcome [8,18,22-25]. In other words, maternal age appeared to be an independent obstetric risk factor for early onset of preeclampsia and impaired fetal growth. It has also been stated that chronic and pregnancy-related hypertension increase risk for low birthweight and preterm birth as well as older maternal age was resulting in shorter gestations [26].

The mechanism behind this risk is beyond the scope of the present study but may be related to aging of the uterine blood vessels [27].

The strength of the current study was that it used a population-based cohort including data from three national Finnish health registries. The data stored in these registries have demonstrated both high quality and validity [28], affirming that the registries are a valuable resource for studying maternal and obstetric health outcomes as the identification of preeclampsia is shown to be accurate in such databases [29]. Weaknesses of the study were that there may have been some error in the data collection, such as coding errors; however, considering the size of the data set, the impact of this factor was probably minor. It is also possible that picking out preeclamptic and eclamptic women from the data on the basis of diagnoses and hospital stay may have slightly increased the number in the sample. However, the data was recorded using a standard format among the registries and therefore, any inconsistency in reporting would have been a random event independent of maternal age.

## Conclusions

In this study, women of AMA had preeclampsia more often than younger women, and their pregnancies were more likely to be complicated by preterm deliveries and impaired fetal growth. As a consequence, the need for neonatal intensive care was significantly higher for women of AMA, suggesting a substantial increase in the use of healthcare resources. Premature infants are at higher risk of neonatal mortality and morbidity as well as neurodevelopmental impairments; these tend to be inversely proportional to gestational age [30]. The preventive measures, such as taking supplements of vitamins C and E, do not reduce the occurrence of preeclampsia [31] but it has been demonstrated that low-dose aspirin started in early pregnancy may reduce the incidence of preeclampsia and intrauterine growth restriction [32]. However, postponing pregnancy to an older age is an obvious risk and the implications for the timing of assisted reproduction and family planning policies are relevant.

## Competing interests

The authors declare that they have no competing interest.

## Authors’ contributions

RL conceived the study with contributions from SH and KVJ. RL prepared the data and performed the statistical analysis. RL and SH interpreted the results with contributions from KVJ and MG. RL reviewed the literature and wrote the manuscript. SH, KVJ and MG critically revised the manuscript for scientific quality and content. All authors approved the final version for publication.

## Pre-publication history

The pre-publication history for this paper can be accessed here:

http://www.biomedcentral.com/1471-2393/12/47/prepub
